# Outcomes of Early Transition of Low-Risk Thyroid Cancer Patients from Specialist to Primary Care

**DOI:** 10.3390/curroncol29100606

**Published:** 2022-10-14

**Authors:** Patricia Nguyen, Parsa Azizi-Mehr, Carol Townsley, Afshan Zahedi

**Affiliations:** 1 Peter Gilgan Centre for Women’s Cancers, Women’s College Hospital, Toronto, ON M5S 1B2, Canada; 2 University of Toronto, Toronto, ON M5S 1A1, Canada; 3 After Cancer Treatment Transition (ACTT) Program, Women’s College Hospital, Toronto, ON M5S 1B2, Canada; 4 Thyroid Program, Women’s College Hospital, 76 Grenville Street, Toronto, ON M5S 1B2, Canada

**Keywords:** thyroid cancer, endocrinology, clinical pathways, transitional care

## Abstract

Background: Recently published clinical pathways for management of thyroid cancer outlined the criteria for transitioning low-risk patients to primary care within one to five years from diagnosis. However, discharge patterns among endocrinologists remain heterogeneous as there lacks a consensus regarding post-treatment care for thyroid cancer patients. Objective: This study described general characteristics and outcomes of thyroid cancer patients who were discharged from specialist care and transitioned to a primary care-based follow-up clinic. Methods: Thyroid cancer patients seen in the After Cancer Treatment Transition (ACTT) clinic at Women’s College Hospital (Toronto, Canada) were included in the study. Electronic medical records were reviewed between May and October 2021 to collect patient characteristics and outcomes. Descriptive statistics were calculated. Results: The study cohort included 148 thyroid cancer patients and 76% were female. All cases were papillary thyroid cancer and most diagnoses were classified as T_2_ (42%), N_0_ (55%), M_0_ (91%), and stage 1 (83%). Nearly all patients (*n* = 147) had complete thyroidectomy. Levels of thyroglobulin and thyroglobulin antibodies (TgAb) were low overall, with only 5% of the study cohort deemed TgAb positive. Mean levels of thyroid stimulating hormone (TSH) measured at 2 time points (1.37 mIU/L, 1.42 mIU/L) were within normal range. About 91% of the study cohort had normal TSH levels and 82% met target TSH levels. There were 2 cases of recurrence; however, investigation determined that they were not initially appropriate candidates for transition to primary care. Nearly 99% (*n* = 146) of patients had excellent response to therapy, showed no evidence of disease recurrence, and have not required re-referral to specialist care. Conclusions: These findings may reassure specialists that low-risk, stable thyroid cancer patients can be safely transitioned to primary care for post-treatment follow-up.

## 1. Introduction

An estimated 8200 Canadians are diagnosed with thyroid cancer each year and it is the fifth most common cancer in Canadian women [1]. The 5-year survival rate for thyroid cancer is one of the highest at 98% [1]. Over 80% of thyroid cancer patients present with well-differentiated thyroid cancer which includes papillary and follicular tumours [2]. After initial surgery and adjuvant treatment, these patients are often followed indefinitely by an endocrinologist who monitors for cancer recurrence through periodic blood tests and neck ultrasounds [3]. However, with the rising incidence of thyroid cancer, the increased need for post-treatment follow-up among survivors, and the limited number of thyroid cancer specialists; there is the opportunity for primary care physicians (PCP) to play a larger role in providing follow-up care. Most of the literature on transitioning care comes from breast and colorectal cancers, where the role of the PCP in long-term follow-up is well established [4,5,6]. There is a lack of research and literature, in general, on the role of PCPs in thyroid cancer follow-up. Cancer Care Ontario recently published clinical pathways for thyroid cancer treatment and outlined the criteria for transitioning low-risk patients to primary care within one to five years from diagnosis [7]. However, discharge patterns from specialist care remain heterogeneous as there lacks a consensus among endocrinologists regarding post-treatment care for thyroid cancer patients [8]. Long-term follow-up by an endocrinologist remains the most common practice.

The After Cancer Treatment Transition (ACTT) program at Women’s College Hospital (Toronto, ON, Canada) was established in 2010, in collaboration with the Princess Margaret Cancer Centre (Toronto, ON, Canada). ACTT is a primary care-based clinic that provides cancer patients with post-treatment follow-up and survivorship care which includes management of therapies, management of late or persisting side-effects, surveillance for recurrence, planning for transition to local PCP, psychosocial support, and linkages to community resources. Referrals to ACTT are based on several criteria, including evidence-based standardized guidelines for follow-up for the cancer type, response to treatment, and oncologist/specialist assessment (i.e., patient health status deemed low risk and stable). Overall, the goals of ACTT are to safely transition cancer patients out of active treatment environments (i.e., hospitals, specialist care), to effectively provide follow-up care, and to appropriately address the needs of cancer survivors. Nearly 75% of the ACTT patient population are breast cancer patients, while the other patients include those with thyroid cancer, gynecological cancer, colorectal cancer, and melanoma. The ACTT program provides comprehensive care through the collaborative efforts of the general practitioner (GP) and the advanced practice nurse (APN). Considering the transitional model of care is not yet well established in thyroid cancer, it is of interest to explore the clinical characteristics and outcomes of thyroid cancer patients who were discharged from specialist care and followed up in the primary care clinic. The purpose of this study was to describe general characteristics and outcomes of thyroid cancer patients followed up in the ACTT program.

## 2. Methods

### 2.1. Study Design and Study Participants

This study was designed as a retrospective, observational study. Any thyroid cancer patients currently or previously followed-up in the ACTT program after thyroid cancer treatment were considered potentially eligible for the study cohort. Patients were excluded if they were referred to ACTT but never had a first visit. A list of potentially eligible patients was provided by the GP and APN in ACTT. Selection bias was minimal as exclusion was based on only one factor which was the patient not having an actual visit to the ACTT clinic, otherwise all thyroid cancer patients seen in ACTT were included. This study was reviewed and approved by the Research Ethics Board of the Women’s College Research Institute.

### 2.2. Data Collection and Analysis

Generally, patients in ACTT are scheduled for annual follow-up visits, thus data was collected for the first ACTT visit and the latest documented follow-up for the patient. Review of electronic medical records was conducted at Women’s College Hospital between May and October 2021 to collect the following clinical variables:

1. Age and sex;
2. Age at thyroid cancer diagnosis;
3. Thyroid cancer pathology;
4. Surgery and treatment;
5. Time between diagnosis and first ACTT visit;
6. Standard blood test levels at first visit and most recent follow-up in ACTT: thyroglobulin (Tg), thyroglobulin antibodies (TgAb), thyroid stimulating hormone (TSH), free thyroxine (T4), and calcium;
7. Any recurrence or other cancers detected;
8. Any information related to discharge from ACTT.

Data extraction was conducted by one member of the research team and data was reviewed for completeness and discrepancies by 2 members of the team. The investigators (an endocrinologist and the ACTT GP) provided clinical expertise and support. TNM classifications and staging were extracted from medical records as documented; however, when verification was needed, it was based on the American Joint Committee on Cancer 8th Edition Staging System, as endorsed by the American Thyroid Association. As a general guideline, patients were described as “low-risk” based on thyroid cancer diagnosis (i.e., TNM classification, staging), response to treatment, post-treatment standard blood test levels, and clinical notes in medical records.

Descriptive statistics including means, proportions, and ranges were calculated. Two-tailed *t*-test was used to compare ages between males and females, with statistical significance at *p* < 0.05.

## 3. Results

Initially, 162 thyroid cancer patients were identified as having been referred to ACTT, and from this list, 148 patients were determined eligible for the study as they had a first visit and received follow-up care in the ACTT clinic. General reasons for not having a first ACTT visit included cancellation of the referral by the specialist, patient moved residence, and patient preference to receive follow-up care immediately by local PCP. At the time of data collection, the mean age of the 148 study participants was 55 years, and the mean age at cancer diagnosis was 43 years (Table 1). The majority of study participants were female (76%, *n* = 113) and the mean age at cancer diagnosis was younger for females (43 years) compared to males (48 years), with statistical significance (*p* = 0.037) (Table 1). The mean time between diagnosis and transition to the ACTT clinic was 4.5 years, which was consistent between males and females (Table 1).

All diagnoses were papillary thyroid cancer, and nearly all cases had no documented vascular invasion (92%, *n* = 136) and no documented extra thyroidal extension (95% *n* = 141). The mean tumour size was 2.45 cm and this was consistent between males (mean size 2.45 cm) and females (mean size 2.46 cm). In terms of TNM and cancer stage classifications, most cases were T_2_, N_0_, M_0_, and stage 1 thyroid cancer (Table 1). All patients had surgical treatments, with 68% (*n* = 101) undergoing total thyroidectomy, compared to 31% (*n* = 46) who had thyroid glands removed in a 2-stage process (Table 2). The majority of patients received additional treatment with radioactive iodine (64%, *n* = 95) (Table 2). As per standard follow-up guidelines, nearly all patients in the study cohort (95%, *n* = 140) had head and neck ultrasounds.

Standard follow-up also includes monitoring levels of Tg (non-stimulated), TgAb, TSH, free T4, and calcium. Mean levels for Tg and TgAb were overall low at the initial ACTT visit (Tg: 0.64 µg/L; TgAb: 18.87 KIU/L) and at the latest documented ACTT follow-up (Tg: 0.53 µg/L; TgAb: 15.11 KIU/L) (Table 3). The proportion of the study cohort deemed TgAb positive (>20 KIU/L) was 5% (*n* = 8). Mean levels for TSH, free T4, and calcium were within normal range at both time points (Table 3). About 91% (*n* = 134) of the study cohort were within normal TSH range (0.5–4.5 mIU/L) at the initial ACTT visit and 86% (*n* = 127) were within range at the latest documented ACTT follow-up. Generally, for low-risk patients, the target TSH level is between 0.5 to 2.5 mIU/L, and 82% (*n* = 122) of the study cohort were within this target range at the first ACTT visit and 77% (*n* = 114) were within this target range at the latest ACTT follow-up.

Besides thyroid cancer, 16% (*n* = 23) of the study cohort had other cancer diagnoses which mostly occurred years before their care was transitioned to ACTT; however, there were 2 patients who were diagnosed with other cancers while already in ACTT. These other cancer diagnoses included breast cancer, cervical cancer, stomach cancer, colon cancer, melanoma, and sarcoma. There were 2 cases (1.3%) of thyroid cancer recurrence. Both cases were documented to have lymph node metastasis and high levels of TgAb prior to their initial visit in ACTT, including one case with an extremely high level of TgAb (>1000 KIU/L). After both patients were re-referred to specialist care, one case did transition back to the ACTT clinic and has since been discharged to their local PCP. Overall, 53% (*n* = 79) of the study cohort have been discharged from ACTT and fully transitioned to their local PCP, with most cases documented as stable and at least 5-years post-diagnosis. The remaining thyroid cancer patients have not yet been formally discharged from ACTT, although to date, no patients have required re-referral to specialist care as no recurrence has been identified. Any patients eligible for further transition to their local PCP will be considered for discharge from ACTT.

## 4. Discussion

The current study provided a rare opportunity to explore the clinical characteristics and outcomes of thyroid cancer patients who were discharged from specialist care and followed up in a post-treatment clinic led by a GP. Based on the pathology and blood test levels collected from electronic medical records, the study cohort was overall a low-risk, stable patient population, with excellent response to therapy, that met the criteria for discharge from specialist care and for ongoing follow-up in a primary care clinic. It was expected that the study cohort consisted of stable patients considering the average time since thyroid cancer diagnosis was 4.5 years. Overall, recurrence was very low (1.3%) and the 2 cases of recurrence had incomplete response to therapy. Therefore, they did not initially meet the criteria for discharge from specialist care and were likely not appropriate candidates for transition to the ACTT clinic. All other patients with complete response to therapy showed no evidence of disease recurrence. It was encouraging that the majority of thyroid cancer patients in our cohort have been safely discharged from the ACTT clinic and effectively transitioned to their local PCPs. To date, no patients in our cohort have required re-referral back to specialist care.

As noted earlier, the transitional model of care is not well established in thyroid cancer. There lacks a consensus among endocrinologists regarding post-treatment care of thyroid cancer patients despite updated guidelines which recommend the transition of low-risk patients to PCPs within 1 to 5 years from diagnosis [7,8]. Previous literature on thyroid cancer survivors have shown that possible patient barriers to transitioning care included concerns related to side effects of thyroid hormone fluctuation, fear of disease recurrence, and a lack of confidence in PCPs to manage thyroid cancer follow-up, including unmet psychosocial needs [9,10,11,12]. A recent study investigated how PCPs reported their involvement in thyroid cancer survivorship care [13]. Its findings indicated that there was a wide range in reported involvement, with most (34%) reporting “sometimes” involvement and only 24% and 18% reporting “often” and “almost always” involvement, respectively. Another recent study examined long-term healthcare utilization after childhood or young adult thyroid cancer, and one of its findings demonstrated that even after a substantial number of years post-diagnosis (i.e., 20 years), follow-up care was predominantly provided by endocrinologists compared to PCPs [14]. Overall, findings from these studies further suggest that it is not standard of care for endocrinologists to discharge stable thyroid cancer patients and transition them to PCPs for ongoing follow-up and survivorship care.

However, the literature does present reassuring evidence that post-treatment follow-up and survivorship care can be effectively provided in primary care. Previous studies in breast cancer have shown that exclusive PCP follow-up was not associated with delay in diagnosing recurrence or an increased rate of serious clinical events [15,16]. Other findings suggested that the quality of life for breast cancer survivors improved when PCPs assumed greater responsibility for ongoing care [6,17]. Furthermore, PCPs are known to be well experienced in the management of breast cancer comorbidities, including dyslipidemia and depression [15,16,18,19]. Transitioning patients to primary care is also beneficial to the healthcare system, especially to ease the strain on specialists and hospital resources. A recent study comparing primary versus tertiary follow-up care of low-risk differentiated thyroid cancer suggested that follow-up in primary care provided significant economic benefits to the healthcare system [20]. The findings by Imran et al. also demonstrated that recurrence rate remained low in primary care and a significantly high proportion of patients met target TSH levels, which is consistent with the results of our study [20]. Lastly, follow-up in primary care may also be more accessible and sustainable for cancer survivors. It may be more feasible to see a physician in the local community compared to seeing a specialist in a hospital or large cancer center. The ACTT clinic functions as an “intermediate” between specialist and primary care and in turn, it promotes the continuity of care, ensures the accurate exchange of information from specialist to PCP, and prepares both patient and PCP for a smooth transition of care, especially to reduce any anxiety or lack of confidence. However, it is important to remember that the ACTT clinic is led by a GP; therefore, its approach for transitioning patients and delivering follow-up care can be adopted by other PCPs in community practices. Moura et al. described in detail the development and implementation of the ACTT program, including the inter-professional clinical care, the application of standardized guidelines for follow-up care, the use of survivorship care plans, and the efforts to address psychosocial needs of cancer survivors [21].

It is worth noting that there are limitations to this observational study. All data was collected from patient electronic medical records which relies on the physician or clinical staff to input the medical history, clinical notes, and relevant imaging or blood test results. There may be some degree of variation and inconsistency in clinical documentation among the different physicians and clinical staff. However, two members of our research team reviewed the collected data for completeness and discrepancies, using a systematic and consistent approach, and the study investigators were consulted on missing or questionable data. Another limitation was our small study cohort which consisted of 148 thyroid cancer patients. The small sample size limited comprehensive analysis of patient characteristics and outcomes (i.e., subgroup analyses). However, considering that the transitional model of care remains not yet well established for thyroid cancer, this is one of the few studies available to examine a cohort of thyroid cancer patients who were discharged from specialist care and transitioned to ongoing follow-up with a GP. It is worthwhile to note that our findings have the potential to represent other similar thyroid cancer patient populations, particularly since the ACTT clinic is of service to patients from Toronto and neighbouring cities, all of which are fairly large cities (Toronto has >2 million people) with substantial diversity. Furthermore, the ACTT clinic provides follow-up care based on current evidence-informed recommendations and guidelines from prominent health organizations such as American Thyroid Association and Cancer Care Ontario. However, there is always the need for further research, and this includes conducting a more comprehensive examination of patient outcomes (i.e., symptoms, side effects, and psychosocial needs) and assessing the experiences of patients and PCPs as it relates to the transition of care and long-term follow-up (i.e., perceptions, satisfaction level, positive and negative factors).

## 5. Conclusions

The current study demonstrated that low-risk, stable thyroid cancer patients who responded well to treatment, can safely be discharged from specialist care and effectively transitioned to a primary care physician for ongoing follow-up. These findings may provide endocrinologists with more confidence and reassurance in transitioning thyroid cancer patients post-treatment to primary care.

## Figures and Tables

**Table 1:** Clinical characteristics of diagnosis for thyroid cancer patients in After Cancer Treatment Transition (ACTT) clinic.

		Total, *n* = 148	Female, *n* = 113	Male, *n* = 35
Mean age, in years (range) ^a^		55 (29–80)	54 (29–78)	59 (36–80)
Mean age at diagnosis, in years (range) ^b^		43 (18–70)	43 (18–70)	48 (29–69)
Mean time between diagnosis and first ACTT visit, in years (range)		4.5 (1–15)	4.6 (1–15)	4.3 (1–11)
		Classification		*n* (%)
TNM	Tumour site (T) ^c^	T_1_		26 (18)
		T_1a_		16 (11)
		T_1b_		21 (14)
		T_2_		62 (42)
		T_3_		16 (11)
		T_3a_		3 (2)
		T_3b_		1 (<1)
		T_4_		1 (<1)
	Lymph nodes (N) ^c^	N_0_		81 (55)
		N_1_		11 (7)
		N_1a_		30 (20)
		N_1b_		10 (7)
		N_x_		14 (9)
	Metastatic status (M) ^d^	M_0_		134 (91)
		M_x_		11 (7)
Staging ^d^		1		123 (83)
		2		19 (13)
		3		3 (2)

^a^ No significant difference in current age between males and females, *p* = 0.051. ^b^ Significant difference in age at diagnosis between males and females, *p* = 0.037. ^c^ Information not available for 2 participants. ^d^ Information not available for 3 participants.

**Table 2:** Thyroid cancer treatments.

	Treatment	*n* (%)
Surgery	Total thyroidectomy (with or without neck dissection)	101 (68)
	2-stage thyroidectomy	46 (31)
	Hemithyroidectomy	1 (1)
Radioactive iodine (RAI) ^a^	Yes	95 (64)
	No	53 (36)

^a^ Median RAI dose was 50 mci (range 29–150 mci).

**Table 3:** Blood test levels for thyroid cancer patients in After Cancer Treatment Transition (ACTT) clinic.

	Normal Levels a	Mean Level at First ACTT Visit (Range)	Mean Level at Latest ACTT Visit (Range)
Thyroglobulin (Tg), µg/L ^b^	<0.9	0.64 (0.04–4.5)	0.53 (0.01–18)
Thyroglobulin antibodies (TgAb), KIU/L ^b^	<20	18.87 (10–156)	15.11 (8.84–92)
Thyroid stimulating hormone (TSH), mIU/L	0.5–4.5	1.37 (0.01–18.14)	1.42 (0.01–15.9)
Free thyroxine (T4), pmol/L	7.72–15.45 (Normal for women is 10–20)	19.17 (2.31–33)	19.49 (4.17–33)
Calcium, mmol/L	2.2–2.6	2.52 (1.26–2.6)	2.32 (0.25–2.56)

^a^ As indicated on laboratory reports and based on how the blood tests were designed. ^b^ For laboratory results that were reported as an indefinite value (i.e., <0.9), the numerical value was used in the mean calculations.

## Data Availability

The data presented in this study is available on request from the corresponding author.
